# Embodied behavioural complexity in a ciliated microorganism

**DOI:** 10.1038/s41467-026-75076-8

**Published:** 2026-07-08

**Authors:** Alexander K. Boggon, Alasdair D. Hastewell, Jörn Dunkel, Kirsty Y. Wan

**Affiliations:** 1 Living Systems Institute & Department of Mathematics and Statistics, University of Exeter, Exeter, UK; 2 NSF-Simons National Institute for Theory and Mathematics in Biology, Chicago, IL, USA; 3 Department of Mathematics, Massachusetts Institute of Technology, Cambridge, MA USA

**Keywords:** Biological physics, Cell migration

## Abstract

Most animals coordinate behaviour using neural computations. Yet, single-celled organisms also exhibit stimulus-responsive, even cognitive, actions. To understand how a single cell can coordinate and drive complex behaviours without any neural encoding, we study an algal protist – a motile cell with four extremely long cilia. The organism displays a surprisingly rich locomotor repertoire, emerging from the intricate dynamics of the cilia, which form a tight bundle when swimming. We use high-speed quantitative live imaging to extract the spectrum of possible ciliary beating patterns and derive a dispersion relation coupling the temporal frequency and spatial wavelength of cilia oscillations. We further reconstruct the manifold embedded in the behavioural space, showing that despite the range and complexity of ciliary beating modes, the underlying behavioural manifold is intrinsically low-dimensional with non-trivial topological structure. Dynamic transitions in motility patterns are encoded as trajectories in this space, thus reducing the macroscopic behavioural states to the underlying microscopic dynamics of the cilia.

## Abstract (web summary)

The complex locomotor behaviours of single-celled organisms are primarily characterised at coarse scales, with limited understanding of how subcellular ciliary dynamics translate into whole-organism movement. Here, the authors report a multi-scale analysis of the ciliary beating patterns in *Pterosperma*, revealing that despite their apparent complexity, these patterns are constrained by a low-dimensional, structured behavioural manifold governed by an empirically derived dispersion relation.

## Introduction

Behaviour is shaped by an organism’s internal state and sensory experiences. Quantitative ethology has helped reveal the deep evolutionary origins of information processing and natural computation in behaving animals^1,2^. In vertebrates, neuronal networks and complex patterns of electrical activity encode sensory inputs, control body posture, and coordinate movement gaits^3,4^. In *C. elegans* crawling, the worm’s neuromuscular control architecture can be derived from dynamical systems representations of the worm’s posture changes^5,6^. In *Hydra*, a cnidarian with a simple distributed nervous system, ‘wireless’ signalling via neuropeptides controls sophisticated behaviours, such as somersaulting^7^. In the context of behaviour, life’s smallest organisms are often overlooked. Yet, despite their minuscule size and relative simplicity, microorganisms routinely perform remarkably complex actions from directional sensing and tactic navigation to prey-capture, feeding, foraging, and even mating^8–10^. All this is achieved without a nervous system or any musculature that we would typically find in animals executing similarly sophisticated tasks. How do we study and quantify complex behaviour at the scale of a single-celled microorganism? And how do these behaviours relate back to the underlying dynamics of the locomotor apparatus? Typically, such studies are hindered by the challenge of imaging behavioural processes at the requisite spatial and temporal resolution. Conventional brightfield microscopy cannot easily resolve subcellular features or motion actuators such as the characteristic shape changes of cilia or flagella, due to their fast dynamics, or intrinsically three-dimensional actuation patterns^11–13^. For example, fluorescent labelling of flagella was needed to visualise how the enteric bacterium *C. jejuni* coordinates two opposing flagella for swimming by wrapping the leading flagellum around the cell body as it swims^14^. As such, behavioural studies of microswimmers have typically focused on coarse-grained descriptions of cell motility trajectories^15–18^, while detailed subcellular dynamics have only been accessible in tethered or otherwise confined cells^19–22^, or in isolated ciliary axonemes^23^. Thus, despite advances in imaging and marker-less tracking^24^, we still lack a tractable experimental system that allows us to bridge the subcellular dynamics of microscopic appendages to whole-organism behaviour.

**Fig. 1:**
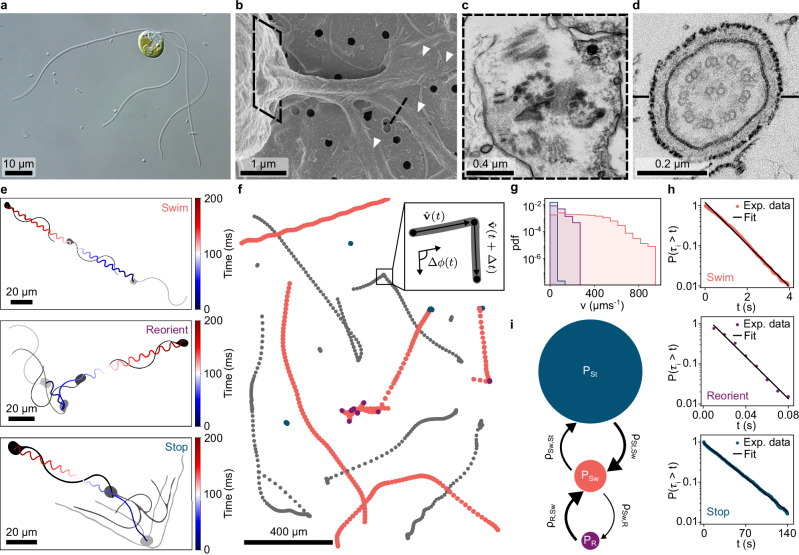
Organism morphology and free-swimming dynamics. **a** *Pterosperma* cell imaged by differential interference contrast microscopy. **b** Scanning electron microscopy image showing all four cilia emanating from the same location within the cell body, white arrows indicate the cilia. **c**, **d** Transmission electron microscopy images (corresponding to the cross sections annotated by the black dashed lines in (**b**)) of the arrangement of basal bodies, striated fibres and ‘9 + 2’ ciliary axonemes. **e** Illustrative cell body trajectories and ciliary arrangements during three distinct behavioural macrostates. From top to bottom: Swim, Reorient  → Swim, Stop  → Swim. **f** Example free-swimming trajectories (see Supplementary Video 1), selected trajectories are coloured by state: Swim (coral), Reorient (purple), Stop (teal). Inset: schematic illustrating cell body velocity *v* and turning angle Δ*ϕ*. **g** State specific cell speed histograms for *n* = 2096 trajectories. **h** State survival probability *P*(*τ*_*i*_ > *t*), coloured circles indicate experimental values, exponential fits are shown with a solid black line. **i** Transition network with statistics obtained from the Markov model. Steady state probabilities $({P}_{{{{\rm{Stop}}}}},{P}_{{{{\rm{Swim}}}}},{P}_{{{{\rm{Reorient}}}}})=(0.966\pm 0.002,0.033\pm 0.001,0.00030\pm 0.00005)$. Probability of each transition (*ρ*_St→Sw_, *ρ*_Sw→St_, *ρ*_Sw→R_, *ρ*_R→Sw_) = (1, 0.70 ± 0.04, 0.30 ± 0.04, 1).

To address this challenge, we establish a motile marine alga (*Pterosperma*) as a powerful model system to investigate embodied behavioural encoding in an aneural single-celled organism. The species, which belongs to the basal lineage of Prasinophyte flagellates, is widely distributed and plays a critical role in marine ecosystems^25,26^. During part of its life cycle, the cells form highly resistant cyst-like structures known as phycomata, which have even been observed in Silurian fossil records^27^. Each *Pterosperma* cell has a 10 *μ*m cell body and four extremely long cilia which emerge from an anterior groove (Fig. 1a). Its complex ciliary apparatus confers a unique form of fast motility, in which all four cilia tightly bundle to function as a single compound cilium^28^. The bundle maintains its integrity during forward swimming and even through diverse transitory manoeuvres. In contrast to organisms whose motion repertoire is exclusively controlled by neural circuits, here, the instantaneous behaviour derives from the shape dynamics of the ciliary bundle itself, a characteristic typical of many organisms that locomote using soft body undulations and embodied computations in the non-inertial regime^29,30^. To capture the full behavioural space of this species, we develop a multi-scale approach spanning population dynamics down to single cilium activity. We leverage the organism’s highly planar ciliary beat waveform, performing high-speed imaging to track and resolve ciliary shape dynamics over millisecond timescales. By decomposing these waveforms into well-defined shape modes and examining continuous transitions in behaviour, we discover that despite the rich waveform dynamics and apparent behavioural complexity, the beat patterns are constrained by an empirically determined dispersion relation, and are captured by a surprisingly low-dimensional phenotypic space. This unique dataset, obtained for the first time from a living organism, highlights not only the extent to which cilia can reversibly modulate their beating dynamics all the way from quiescence to large amplitude oscillations, but also the temporal connectivity between different beating modes.

Across multiple eukaryotic taxa, groups of cilia routinely self-assemble and organise into myriad specialised structures that mediate complex functions from sensing to motility, with minimal or no centralised control^31,32^. Given the highly conserved nature of the cilium across eukaryotes, we ask, what is the space of all possible beating dynamics on a single motile cilium? Our work uncovers the geometric encoding of gaits and behaviours that are driven by ciliary beating. In particular, our analysis reveals a vast spectrum of functional ciliary waveforms existing on the same structure, enriching our perspective on how dynein motors distributed along a cilium self-organise to produce emergent dynamics^33,34^. Taken together, we provide empirical evidence for how a single celled organism’s distinctive swimming behaviours are not only physically embodied in the shape dynamics of the cilia actuators themselves, but also fundamentally constrained by the nanoscale motor mechanics underlying ciliary beat generation.

## Results

### Quantification of free-swimming behaviour

The freely swimming *Pterosperma* cells studied here have a distinctive morphology consisting of an ellipsoidal cell body with dimensions (9 ± 1) × (7.1 ± 0.8) *μ*m, and four cilia that emerge from an anterior groove (Supplementary Fig. 1a). The cilia are unusually long (Fig. 1a), each with a typical length of 67 ± 6 *μ*m (Supplementary Fig. 1b). Both body and cilia are covered with scales, which form a primitive cell wall^25,28,35^. We visualised the ciliary apparatus using scanning and transmission electron microscopy to reveal that the cilia are anchored within the cell body by basal bodies that assume a 3 + 1 arrangement^28^ (Fig. 1b). Fibrous structures connecting the basal bodies likely mediate cilia bundle synchrony, as shown in other microalgae^20^ (Fig. 1c). All cilia have the highly conserved 9 + 2 axonemal structure (Fig. 1d).

We identified three behaviours: long periods in which the cell body remains stationary (Stop), followed by smooth forward swimming (Swim) interrupted by rapid reorientation events (Reorient). The organism typically unfurls its four cilia in the Stop state (Fig. 1e), but always bundles them as a single ‘compound cilium’ during swimming and reorientations. Ciliary bundle formation is therefore temporary and arises from hydrodynamic and steric interactions; indeed, no physical connective components have been observed between the cilia except between the basal bodies^28^. In the Swim state, a travelling wave propagates down the cilium to drive forward propulsion while reorientations are achieved through large curvature bends of the compound cilium (Fig. 1e).

To reveal the divergent scales over which the three main behaviours are performed, we captured thousands of single-cell trajectories across the population using a 10 × objective at 100 frames per second (fps) (Fig. 1f & Supplementary Video 1). Cells were imaged at 10 − 20 *μ*m above the glass substrate. Each state in our tripartite classification is automatically identified based on the instantaneous swimming speed *v*(*t*) and the cosine of the turning angle Δ*ϕ* ∈ [ − *π*, *π*], $\cos \Delta \phi (t)=\hat{{{{\bf{v}}}}}(t)\cdot \hat{{{{\bf{v}}}}}(t+\Delta t)$ (Supplementary Fig. 2), where $\hat{{{{\bf{v}}}}}(t)={{{\bf{v}}}}(t)/\left\vert {{{\bf{v}}}}(t)\right\vert$. The speed distribution (Fig. 1g) is broad, with *v* ≤ 20 *μ*m s^−1^ corresponding to Stop states. Reorientations are identified as epochs where $\cos \Delta \phi < 0.99$ (Supplementary Fig. 2) and the swimming temporarily slows down.

This procedure automatically classifies the motility state as *i* ∈ {Stop, Swim, Reorient} across *n* = 2096 single-cell trajectories and 3947 transitions, to yield a robust quantification of the overall behaviour. State transitions can be modelled as a continuous time Markov chain (Supplementary section B.2), which admits the simple network structure shown in Fig. 1i. We calculated the mean state lifetimes 〈*τ*_*i*_〉, the steady state probability *P*_*i*_ of finding the cell in state *i*, the transition probabilities *ρ*_*ij*_ for the cell to switch from state *i* to state *j*, as well as the associated transition rate *q*_*ij*_. State survival probabilities *P*(*τ*_*i*_ > *t*) = *N*(*τ*_*i*_ > *t*)/*N*(*τ*_*i*_ > 0) (the probability for a cell to remain in a state for a time longer than *t*)^16,36^ decay approximately exponentially (Fig. 1h).

The three behavioural motifs occur over distinct timescales. Mean state lifetimes span four orders of magnitude in time from the highly persistent Stop state (〈*τ*_*St*_〉 = 58 ± 2 s), through swimming (〈*τ*_*Sw*_〉 = 1.42 ± 0.03 s), to the extremely short lived reorientations (〈*τ*_*R*_〉 = 0.041 ± 0.002 s). Only specific transitions are allowable in this coarse-grained behavioural space: no Stop ↔ Reorient transitions are ever observed, therefore passage between these states must occur via the Swim state. We estimate the state probabilities $P(t\to \infty )=({P}_{{{{\rm{Stop}}}}},{P}_{{{{\rm{Swim}}}}},{P}_{{{{\rm{Reorient}}}}})=(0.966\pm 0.002,0.033\pm 0.001,0.00030\pm 0.00005)$, so that the cell is extremely likely to be in the Stop state, possibly to conserve energy (see Discussion). We can also compute the probability of transitioning between each state (*ρ*_St→Sw_, *ρ*_Sw→St_, *ρ*_Sw→R_, *ρ*_R→Sw_) = (1, 0.70 ± 0.04, 0.30 ± 0.04, 1), noting that *ρ*_Sw→St_ + *ρ*_Sw→R_ = 1. The transition rates (*q*_St→Sw_, *q*_Sw→St_, *q*_Sw→R_, *q*_R→Sw_) = (0.0174 ± 0.0005, 0.50 ± 0.01, 0.21 ± 0.01, 24 ± 1) s^−1^ fully characterise the motility dynamics.

### Active ciliary bundle oscillations drive distinct behaviours

To understand how these distinct behaviours are produced by the cell’s unique morphology and cilia dynamics, we performed high-magnification differential interference contrast imaging of single-cell swimming behaviour at 1000 fps using a 40 × and 63 × objective to capture ciliary bundle and single-cilium dynamics, respectively. These high spatiotemporal resolution videos are used to resolve the intricate dynamics of the ciliary apparatus during each of the three behavioural macrostates (Supplementary Video 2).

**Fig. 2:**
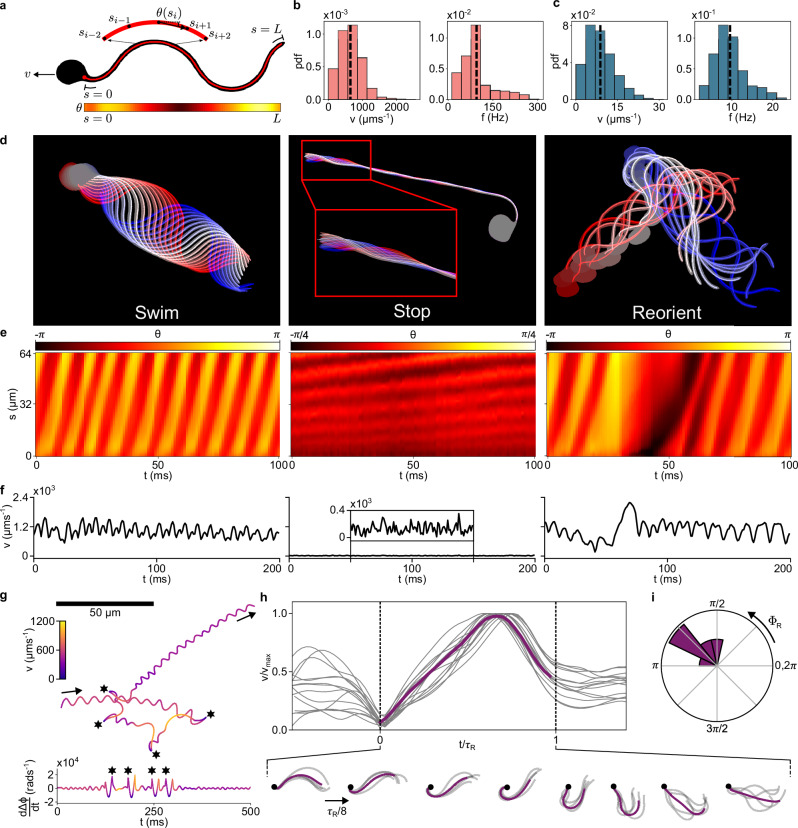
Ciliary bundle displays complex waveforms. **a** Ciliary (bundle) waveform parametrised by tangent angle and arc length *θ*(*s*). **b**, **c** Histograms of cell body speed (left) and ciliary beat frequency (right) during Swim (n_cells_ = 92) and Stop (n_cells_ = 14, n_cilia_ = 16) states. Dashed black line indicates mean value. $\langle {v}_{{{{\rm{Swim}}}}}\rangle=646\pm 326\,\mu {{{\rm{m}}}}\,{{{{\rm{s}}}}}^{-1}$, $\langle {f}_{{{{\rm{Swim}}}}}\rangle=95\pm 53\,{{{\rm{Hz}}}}$ and 〈*v*_Stop_〉 = 9 ± 5 *μ*m s^−1^, 〈*f*_Stop_〉 = 10 ± 4 Hz (mean  ± s.d.) **d** Cell silhouette with cilium trace overlaid and colour coded by time, for each behavioural macrostate. **e** Kymographs of *θ*(*s*, *t*) (arc length *s* and time *t*) corresponding to each behaviour above. **f** Sample timeseries of cell speed *v*(*t*) in each state. **g** Trajectory of cell undergoing sequential reorientations (top) with corresponding angular speed profile (bottom). Asterisks indicate reorientations. **h** Cell speed profiles, normalised by the max speed ${v}_{\max }$ and reorientation time *τ*_R_, show reorientation dynamics are stereotyped (n_cells_ = 11, n_reorientations_ = 14). Inset: Phase-ordered waveforms traced during reorientations (thick lines: waveforms averaged across 4 cells). **i** Circular histogram of reorientation angle Φ_*R*_ (n_cells_ = 11, n_reorientations_ = 14).

Ciliary waveforms were extracted from each frame using custom MATLAB^®^ code leveraging the medial axis transform^37^ to delineate between the cell body and cilium proper. We focus on the dynamics of the centreline of the ciliary bundle, fully described by its instantaneous tangent angle *θ*(*s*, *t*) parametrised by arc length *s* and time *t* (Fig. 2a., Supplementary section C). Distinct ciliary beating patterns underlie the highly variable cell propulsion modes observed in Fig. 2d.

In the Swim state, the cilia remain bundled. Robust base-to-tip travelling waves propagate with highly variable frequency and amplitude. The distribution of frequencies ranges from 12 to 304 Hz with a mean of 95 Hz, resulting in swimming speeds varying across two orders of magnitude (Fig. 2b) (n_cells_ = 92). Such a broad spectrum contrasts with previous measurements from other organisms and sperm flagella, where cilia beat frequencies are typically more tightly defined for any given species^21,38–40^ (Supplementary Table 1).

The Stop state is associated with minimal body movement yet active cilia oscillations (Fig. 2c). Here, robust base-to-tip directed waves are observable on the distal 3/4 of the cilium, with mean beat frequencies of 10 ± 4 Hz (Fig. 2c, d) (n_cells_ = 14, n_cilia_ = 16). However, due to the randomised orientation of the unbundled cilia, the cell motion resulting from these cilia oscillations is largely non-progressive, with average cell migration speed 8.83 ± 0.01 *μ*m s^−1^ and diffusivity of only 0.2 *μ*m^2^ s^−1^ (Supplementary Section B.3). The functional role of this novel oscillatory mode of motile cilia remains unclear but may be to enable active sensing^16^.

Finally, the Reorient mode is detected as an abrupt change in the swimming trajectory and speed (Fig. 2g). This results from rapid changes in the ciliary bundle waveform (which remains intact), in contrast to the quasi-steady oscillations displayed by the cilia during the Swim and Stop states (Fig. 2d–f). Aligning 14 reorientations from 11 cells, we found them to be highly stereotyped (Supplementary Fig. 5). Typically, a large initial bend develops at the cilium base, which rapidly brings the cell to a halt, rotating the cell body through  ~ 180^∘^. This bend propagates down the cilium propelling the cell rapidly forward, followed by a relaxation back into the Swim state, during which waveform stereotypy temporarily breaks down. We estimate that reorientations produce a stereotyped turning angle peaked around 130 ± 30^∘^ with a duration of 36 ± 3 ms (Fig. 2i & Supplementary Fig. 5). Reorientations are an essential component of the organism’s tripartite navigation strategy, akin to ‘tumbles’ in other microswimmers^17,41^.

### High-resolution spectral decomposition of ciliary beating modes

So far, we have established that *Pterosperma* displays many distinct behaviours driven by stereotyped dynamics of their cilia. To reveal the temporal evolution of different ciliary oscillation patterns, we seek a unifying framework capable of capturing this extraordinary complexity using a common shape basis for all observed beat waveforms. Methods previously employed to capture shape information from undulatory locomotion based on PCA or Fourier modes are either non-differentiable or cannot adequately resolve the highly transient, non-oscillatory modes we observe during reorientations. Instead, we decompose ciliary bundle shapes using Chebyshev polynomials of the first kind *T*_*n*_(*s*)^42^. Such a basis is advantageous since Chebyshev expansions converge spectrally for non-periodic smooth functions, allowing for accurate shape reconstruction^43^. They also admit analytical derivatives, and the coefficients are interpretable physically as bending mode amplitudes.

**Fig. 3:**
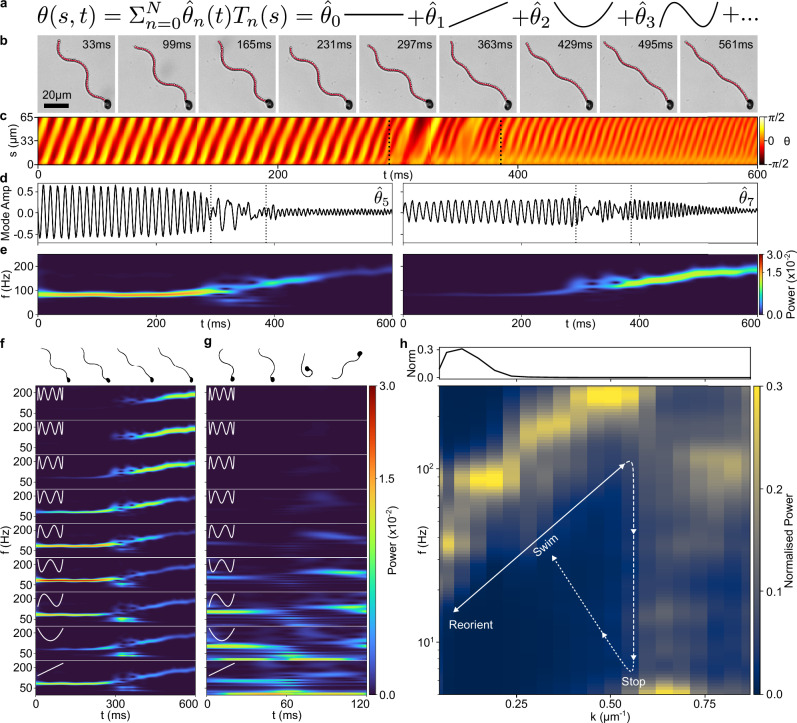
Spectral decomposition of ciliary bundle dynamics. **a** Pictorial representation of the instantaneous ciliary bundle shape as a superposition of Chebyshev modes. **b** Raw video frames of a mode transition sequence overlaid with the Chebyshev waveforms reconstructed with 20 modes. **c** Kymographs of tangent angle representation *θ*(*s*, *t*) (same dataset as **b**). **d**, **e** Selected Chebyshev mode amplitudes ${\hat{\theta }}_{5,7}$ corresponding to the ciliary bundle traces in **b** and their normalised continuous wavelet transforms, ${\hat{{{{\bf{W}}}}}}_{{{\Psi }}}(f,t)$. **f**, **g** Wavelet vectors, modes 1 − 9, for a swimming cell modulating its beat pattern (**f**, same sequence in **b**–**e**) and a cell performing a reorientation (**g**). **h** Dispersion relation *f*-*k* for the entire dataset of *n* = 219, 368 ciliary bundle waveforms, from 125 cells. The magnitude at each *k* (top) is used to normalise the heatmap. The approximate locations of the three behavioural macrostates are labelled. Arrows display the bidirectional and unidirectional movement in the Swim state and Swim *⇌* Stop transitions, respectively.

We express the instantaneous shape of the cilium (or ciliary bundle) as a linear superposition of Chebyshev modes (Fig. 3a.) weighted by their respective time-dependent amplitudes ${\hat{\theta }}_{n}(t)$:

1 $$\theta (s,t)={\sum}_{n=0}^{N}{T}_{n}(s){\hat{\theta }}_{n}(t)\,$$

where the coefficients are determined by first fitting an expansion in Chebyshev polynomials along (*x*, *y*) to determine $\hat{x}(t)$ and $\hat{y}(t)$ using a trust region regularised least squares, motivated by the convergence properties of Chebyshev polynomials for smooth functions. Using the Chebyshev expansions of (*x*, *y*) the curve can then be efficiently re-parametrised to an arclength representation *θ*(*s*, *t*) determined using $\theta (s,t)=\arctan ({\partial }_{s}y(s,t)/{\partial }_{s}x(s,t))$ where the derivatives are calculated from the spectral representation. The theta coefficients are then obtained using Chebyshev projection on *θ*(*s*, *t*). The 0^th^ coefficient of the decomposition corresponds to a weighted instantaneous orientation of the cilium with respect to the lab frame horizontal axis (Supplementary Fig. 4d) and the 1^st^ coefficient to a weighted average curvature, while higher modes describe more detailed cilium shape features. Here we used a total of *N* = 20 modes to capture the entire ciliary shape space with a root mean square reconstruction error of 0.368 *μ*m on average (Supplementary Fig. 4c).

We apply this decomposition to high-resolution sequences of *Pterosperma* cilia. To capture both the dominant shape modes at each time point as well as its time-varying frequency content (Fig. 3b–e), we compute the continuous Morlet wavelet transform ${W}_{\Psi }[{\hat{\theta }}_{n}](a,b)=\frac{1}{a}\int_{-\infty }^{\infty }{{{\rm{d}}}}t\,{\hat{\theta }}_{n}(t){\Psi }^{*}\left(\frac{t-b}{a}\right)$ of the mode amplitudes^44^, for different discrete values of the wavelet frequency scale 1/*a*_*l*_ and time shift *b*_*k*_ = *t*_*k*_, resulting in a wavelet matrix ${({W}_{\Psi,n})}_{l,k}\in {C}^{L,K}={W}_{\psi }[{\hat{\theta }}_{n}]({a}_{l},{b}_{k})$. For every observed ciliary waveform, we can ‘stack’ the wavelets for each mode of the decomposition into a unique ‘wavelet vector’ :

2 $${{{\bf{W}}}}\in {C}^{NL,K}=\left[\begin{array}{c}{W}_{\Psi,0}\\ .\\ .\\ .\\ {W}_{\Psi,N}\end{array}\right]$$

to reveal the wavenumber- and time-dependent frequency information embedded within these complex waveforms. Since the magnitude of the wavelet transform is proportional to the amplitude of an oscillation, we chose to normalise each column of the wavelet vector to have unit norm $\hat{{{{\bf{W}}}}}({t}_{k})={{{\bf{W}}}}({t}_{k})/| {{{\bf{W}}}}({t}_{k})|$, to ensure good resolution across cilia states of varying amplitudes while maintaining the relative importance of each Chebyshev mode within a given state.

We illustrate the results with two very different behaviours. In the first example, a swimming cell is actively modulating the dynamics of its ciliary bundle (Fig. 3b,c). The corresponding wavelet scalograms are plotted for each Chebyshev mode, where ∣*W*_*Ψ*_(*f*, *t*)∣^2^ represents the signal power at each frequency over time (Fig. 3d, e). The time-evolution of wavelet amplitudes reveals unprecedented spectral structure within the swimming state itself (Fig. 3f), allowing us to visualise the rapid but continuous modulation of the large beat amplitude 80 Hz wave to a smaller-amplitude 180 Hz wave. The gradual decay of Chebyshev modes 1 − 5 coincides with the appearance of signal in modes 7 − 9 concurrent with an increasing beat frequency (Fig. 3f). The second example shows a cell undergoing a fast reorientation, whose ciliary dynamics are captured similarly (Fig. 3g). An initially well-defined oscillation is scrambled, with the dominant wavelet amplitudes shifting to the lowest Chebyshev modes. As the cell relaxes back to a new swimming state, the intensity signal percolates from the higher to lower modes before the cell settles into its swimming state again, now dominated by a new, slightly higher beat frequency.

We apply this spectral-wavelet analysis to a large high-resolution dataset of single-cell ciliary dynamics consisting of 219, 368 frames of ciliary bundle shapes from a total of 125 cells. From the instantaneous dynamics of the cilium or ciliary bundle, we can extract dominant temporal frequencies *f* corresponding to a given spatial frequency or wavenumber *k* = 2*π*/*λ* by time-averaging *W*_*Ψ*,*n*_ across cells and assigning to each Chebyshev mode its dominant wavenumber (Fig. 3h).

Our comprehensive dataset captures a remarkable dynamic range in both temporal and spatial frequencies, and occupies two distinct branches in *f*-*k*-space, revealing a ‘dispersion relation’ that constrains ciliary beating. The prominent diagonal band from low to high *f* corresponds to waveforms observed during swimming; cilia transitioning between different beating modes within the Swim macrostate undergo bidirectional movement or fluctuations along this branch (Supplementary Video 3). The swimming dynamics obey an approximately linear dispersion relation with *f* ~ *k* (Supplementary Fig. 6a). The quantisation of the oscillations into four prominent frequency bands, occurring at 37, 88, 184, and 265 Hz (Supplementary Fig. 6b), is a further hallmark of the underlying dynein-driven beat generation mechanism (see discussion below). Meanwhile, the vertical band at low *f* and high *k* corresponds to transitions from the swimming macrostate into the Stop state, accompanied by a dramatic decrease in beat frequency (Supplementary Video 3). Exit from a Stop state is driven by a discontinuous transition in beat mode from a low-*f*, high-*k* state back onto the main diagonal branch.

These sub-cellular dynamics reveal the existence of novel cilia-driven sub-states in the motility repertoire of *Pterosperma* that are completely inaccessible from cell body tracking alone. This also explains the extremely broad distributions of beat frequency and cell speed we measured previously, which do not arise from population heterogeneity but from temporal variability in single cell behaviour.

### Geometric encoding of behaviour on a low-dimensional manifold

We proceed to reconstruct the behavioural state space underlying the entire spectrum of ciliary dynamics a single *Pterosperma* cell can perform, using our extensive dataset of short-duration shape sequences.

**Fig. 4:**
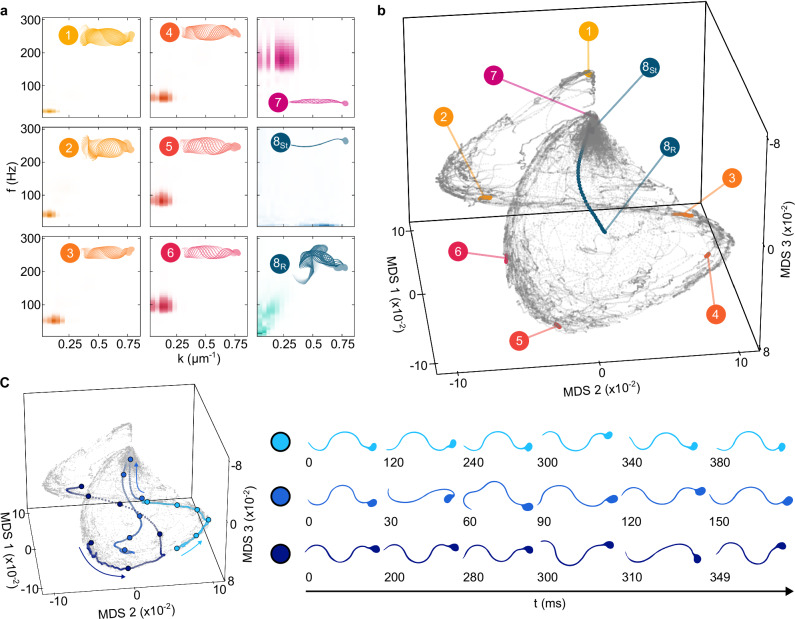
Complex ciliary shape dynamics projected onto a low-dimensional behavioural manifold. **a** Normalised dispersion plots computed for nine illustrative waveform sequences. **b** *Pterosperma’s* behavioural manifold illustrated in the first three MDS dimensions. Grey points indicate the positions on the manifold of all *n* = 219, 368 ciliary bundle waveforms. The manifold coordinates of the waveform sequences in (**a**) are indicated. **c** Three behavioural trajectories on the manifold. Large filled circles indicate the positions of the cell snapshots (right) and arrows the direction of time.

To understand the spatial partitioning of behaviour according to the different modes in Fig. 3h, we identified nine representative waveform sequences and plotted them alongside their corresponding dispersion relations in Fig. 4a. The entire space of beat patterns associated with whole-cell behavioural transitions can be visualised as a single manifold reconstructed from a surprisingly small number of dimensions (Fig. 4b). This is obtained by performing a non-linear dimensionality reduction via multidimensional scaling (MDS) on a distance matrix calculated from the normalised stacked wavelet amplitudes ${d}_{ij}=\left\vert \hat{{{{\bf{W}}}}}({t}_{i})-\hat{{{{\bf{W}}}}}({t}_{j})\right\vert$. Just three MDS dimensions were sufficient to capture 83% of the variance in the data, while six dimensions capture over 95% (Supplementary Fig. 8).

Illustrated here in three MDS dimensions with the nine waveform sequences from Fig. 4a highlighted, the behavioural manifold is two-lobed and highly structured. Following the dynamics along the manifold, we see that beat patterns associated with Swim states define an envelope bounding all other states. Transient behaviours appear as excursions from the main envelope, where the trajectory ventures into the space off the envelope and then rejoins it far from where they left, resulting in a sharp change in the steady swimming behaviour (Supplementary Video 4). These MDS dimensions can be interpreted by colouring the data points by various physical parameters, including cell swimming speed, ciliary beat frequency and wavenumber. This visualisation confirms the general relationship between increasing frequency and high wavenumber, and their co-location with reduced swimming speed (Supplementary Fig. 9). The very dense cluster of points in the centre of the manifold corresponds to the Stop state data and is associated with the lowest speeds.

In this space, two behaviours are considered similar if they correspond to neighbouring points, resolving stereotyped dynamics by distance similarity. For a ciliated single cell, whose motility is dominated by geometric mechanics at zero-Reynolds number^45,46^, there is a clear equivalence between the intrinsic shape dynamics of the cilia and whole-cell behaviour. Our manifold representation provides the basis upon which to bridge embodied whole-organism behaviour to the underlying ciliary actuation mechanism.

For the most part, trajectories flow bidirectionally, following an inverse correlation between beat amplitude and frequency. Yet, nearby phase trajectories can diverge substantially over time, a clear signature of chaotic dynamics (Fig. 4c). In contrast, certain behaviours produce trajectories that only flow in one direction and are therefore irreversible. Reorientations travel into the cluster containing the Stop state, tending to positive values of MDS-1, where the cilia exhibit large deformations. Stop and reorientation events co-localise as each corresponds to very low frequency modes (8_St_ and 8_R_). Meanwhile, cells transitioning towards their Stop state from swimming typically do so by ramping up the oscillation frequency and reducing the amplitude. Such trajectories cross the manifold peak from the negative MDS-1 side and reach a point of no return, beyond which cells are committed to entry into their Stop state. Exiting the Stop state involves a rapid excursion out of the Stop cluster (excitatory phase) back onto the swimming envelope, followed by a refractory period.

## Discussion

Contemporary behavioural studies of microorganisms often target a single scale, either by capturing high-resolution appendage dynamics from immobilised cells^38,47,48^, or using low-resolution centroid-based tracking to follow whole-organism movement^17,41,49^. The latter approach may overlook an organism’s nuanced posture or time-dependent shape dynamics^2,5^. Here, we developed a novel multiscale approach to reconstruct the state-space of all natural behaviours of a single, freely-behaving microswimmer and map them back to the underlying microscopic dynamics of the locomotor appendages.

At the population scale, we adopted a coarse-grained body tracking approach to discretise *Pteropserma’s* behaviour into three states, and mapped the long-time relationships between dominant behavioural modes. The resulting connectivity of the transition network and cellular dynamics in each state suggests a distinct physiological role for each behaviour from energy conservation in the Stop state, through environment exploration during swimming, to reorientations acting as the basis of an escape response.

The various behaviours also exhibited clear timescale separation, from fast reorientations ( < 50 ms), to intermediate timescales of swimming ( ~ 1 s), to the long-lived Stop states ( > 50 s). Our geometric embedding provides new insights into the mechanism of reversible shape transitions over short times and the irreversible dynamics of entry and exit from Stop states over long times. These relationships likely reflect the intracellular control mechanism of ciliary activity, where switching between different ciliary beating modes has been shown in many species to depend on changes in intracellular calcium^50^, ATP concentration^23,51^, membrane electrical potential^32,52^, or cAMP^53,54^.

Coarse-grained behavioural representations intuitively partition macroscopic behaviour into discrete states defined by simple thresholds of swimming or turning speed. However, at the cilium-level motor activity is presumably smoothly controlled. Our discovery of continuous manifold structure in the dynamics of *Pterosperma* ciliary beating supports this hypothesis. By demonstrating how the rich motility dynamics of the organism’s ciliary bundle collapse onto a low-dimensional manifold with striking, well-defined structure, we provide a new framework for exploring and quantifying behavioural complexity in single-celled organisms.

Behaviour exposes the nature of sensorimotor representations in organisms and the prospect of cell-scale cognition. In a soft body, the interplay between deformable morphology and embodied behaviour can effectively orchestrate complex dynamics based on simple actuation rules, applicable to shape-programmable materials^55,56^. Limbless locomotion or undulations of deformable organisms contrasts with traditional neurocentric views of motor control, instead relying more heavily on emergent physical interactions between body morphology, mechanics and the environment^57,58^. To enable fast behavioural reprogramming, many single-celled eukaryotes are also equipped with an excitable cell membrane harbouring an assortment of ion channels that enable threshold behaviours such as ciliary reversal and rapid backward swimming or reorientations^9,52,59^. Dynamical excitability may have been a critical cellular innovation^9^, enabling planktonic algal flagellates to respond rapidly to predation or other sources of mechanical disturbance in the environment^60–62^. For instance, the cilia exhibit small-amplitude oscillations during the quiescent state, suggesting that the system operates close to criticality^16,63^, analogous to hair-cell oscillations^64^: dynamics near a limit cycle rather than a fixed point may enable fast responses, corresponding to rapid excursions on our behavioural manifold (Fig. 4b). A single control parameter, such as concentration of a signalling molecule, could nonlinearly select, for example, via Hopf bifurcation, a wide dynamic range of physical parameters such as beat frequency or waveform without explicit control of dynein function.

Previous studies of ciliary waveform dynamics have typically restricted themselves to single behaviours (such as swimming) and often snapshots of steady state dynamics within them^23,38,65^ (Supplementary Table 1). While these have provided valuable insight into the inner workings of the cilium, alone they give a simplified perspective of the rich dynamics of this key cellular organelle. In contrast, here we set out to map the full spectrum of ciliary beating dynamics generated by a single cell, capturing the fast dynamical transitions between distinct beating modes. In doing so, we uncovered the first empirical dispersion relation for ciliary shape dynamics in any organism: cilia cannot oscillate arbitrarily but are constrained to certain allowable temporal frequencies *ω* = 2*πf* and spatial wavenumbers *k*. This has profound consequences for waveform generation by dynein co-operativity in cilia and (eukaryotic) flagella, which remains incompletely understood^33,34,66–68^. Such a relationship is distinct from and structurally more complex than any observed in other undulatory self-propelled organisms, such as worms or snakes^29,42,69^ (see also Supplementary Fig. 7 for a cross-species comparison of dispersion relations).

To date, analytical models of the ciliary actuation mechanism generally assume small amplitude beating to develop a linear theory with coarse-grained motor kinetics. When fitted to large amplitude ciliary beating^23,70–72^ they predict quadratic and cubic dispersion relations in comparison to our approximately linear *ω*-*k* relationship in the Swim state^70^. Non-linear models which explicitly incorporate dynein kinetics have been developed^40,67^, however these require numerical solvers to predict beat patterns. Such methods are capable of generating a range of large amplitude ciliary dynamics, but predicted functional relationships between sperm number and motor activity cannot be mapped directly to experimentally obtained waveforms and beat parameters.

Looking ahead, future models of ciliary actuation need to be capable of capturing both the full spectrum of beating patterns that a single cell can perform but also reproduce temporal sequences of ciliary beat patterns, not just isolated snapshots of their steady-state dynamics. Physical or biochemical perturbations could be used to determine whether, and to what extent, the mechanochemical constraints that underlie control and actuation of ciliary activity are conserved across eukaryotes.

## Methods

### Cell culture

*Pterosperma sp*. (K-1082, Norwegian Culture Collection of Algae) was cultured by suspending 3 mL of 1-week-old cell culture in 30 mL of TL30 media (https://norcca.scrol.net/node/3956). Cells were grown at 21 ^∘^C and 40 % humidity on a 14: 10 hour light : dark cycle at a light intensity of 2000 lux. Cultures were harvested for experiments 4 − 6 days after inoculation during the late exponential phase of growth at concentrations of  ~ 10^5^ cells/mL.

### Electron microscopy

For electron microscopy studies, cells were fixed in 2% glutaraldehyde and 2% paraformaldehyde in 0.1 M sodium cacodylate buffer (pH 7.2) for 1 h at room temperature. Fixed cells were washed 3 × for $5\,\min$ in the same buffer and post-fixed for 1 h in 1% osmium tetroxide.

For Transmission Electron Microscopy (TEM), following^73^, the post-fixed sample was additionally reduced in 1.5% potassium ferrocyanide in 0.1 M sodium cacodylate buffer.

In both Scanning Electron Microscopy (SEM) & TEM, cells were washed three times for $5\,\min$ each in deionised water before dehydration through a graded ethanol series.

SEM samples were passed through a 200 nm polycarbonate filter using a mild vacuum. After a $3\,\min$ incubation in hexamethyldisilazane [HMDS, Merck, Gillingham, UK], filters were air-dried and mounted onto aluminium stubs with double-sided sticky carbon tabs. Samples were sputter-coated with a 10 nm layer of gold/palladium and imaged using a Zeiss Gemini 500 scanning electron microscope.

TEM samples were gradually infiltrated with Durcupan resin [Sigma Aldrich, Merck, Gillingham, UK] and embedded in BEEM capsules. The resin was polymerised overnight at 60^∘^C. 70 nm sections were collected on pioloform-coated EM copper grids [Agar Scientific, Rotherham, UK] and post-contrasted with Reynold’s lead citrate. Imaging was performed using a JEOL JEM 1400 transmission electron microscope operated at 120 kV, with images captured using a Gatan ES1000W digital camera.

### Brightfield microscopy

Live cell imaging chambers were constructed by spin coating  ~ 0.5 mm Polydimethylsiloxane (PDMS) sheets, punching 6 mm diameter circular wells and securing this section between two cover glass slides.

Imaging was performed 10 − 20 *μ*m above the surface using an inverted microscope [Leica Microsystems DMi8] with a high-speed camera [Phantom Vision Research V1212]. Videos for population analysis were taken under brightfield (BF) illumination at 100 fps using a 10 × (HC PL/0.40) dry objective. For higher-resolution analysis, differential interference contrast (DIC) images were obtained at 1000 fps using a 40 × (HC PL/0.6) dry objective for swimming and reorientation single cell analysis and a 63 × (HC PL/1.2) water immersion objective for cells in their Stop state with a DIC prism providing pixel resolutions of 0.370 *μ*m/pixel and 0.234 *μ*m/pixel, respectively.

### Cilium tracking and reconstruction

A custom cilium tracking protocol was implemented in MATLAB^®^ to extract pixel-wise traces from videos of cell dynamics. Single cell videos are binarised, and the cilium centre line extracted using a threshold on the medial axis transform (MAT)^37^ (Supplementary Fig. 4a).

We approach the parametrisation of the pixel-wise traced ciliary waveform using a shape space decomposition. Here we make use of Chebyshev polynomials of the first kind *T*_*n*_(*s*). These are selected because they are analytically differentiable and effective at capturing non-periodic functions, such as those found in the broad spectrum of ciliary shapes performed by *Pterosperma*, with a small number of modes. Here we provide an overview of the technique; full mathematical details are provided in the supplementary information.

The raw pixel-wise traces **r**(*s*, *t*) = [*x*(*s*, *t*), *y*(*s*, *t*)] can be described by the linear superposition of Chebyshev modes weighted by their time dependent amplitudes ${\hat{{{{\bf{r}}}}}}_{n}(t)=[{\hat{x}}_{n}(t),{\hat{y}}_{n}(t)]$ for *n* ∈ {0, 1…*N*}. The mode amplitudes are obtained with a trust region regularised least squares fit. This requires the constraint that the coefficients should decay exponentially under the assumption that our waveforms are smooth functions^43^ and a regularisation term on the total variation of the second derivative, which enforces smoothness by penalising large changes in the second derivative. In this step, *N* = 125 coefficients are fitted, providing a root mean square error in the reconstruction of less than the size of a pixel on average. Not all coefficients are necessary to describe the shape, but are important for calculating smooth, high-quality derivatives important for re-parametrisation and computing the angular representation.

From the Cartesian space curve correctly re-parametrised by the arc length, a smooth transformation to the tangent angle representation can be achieved:

3 $$\theta (s,t)=\arctan ({\partial }_{s}y(s,t)/{\partial }_{s}x(s,t))={\sum}_{n=0}^{N}{T}_{n}(s){\hat{\theta }}_{n}(t)$$

where the subscript *s* indicates a derivative with respect to arc length.

The Cartesian space representation can be recovered using Equation (4) (Supplementary Fig. 4b):

4 $$\left[\begin{array}{c}x(s,t)\\ y(s,t)\end{array}\right]=x(0,t)+\frac{L}{2}\int_{-1}^{s}ds^{\prime} \left[\begin{array}{c}\cos \theta (s^{\prime},t)\\ \sin \theta (s^{\prime},t)\end{array}\right]$$

where *L* is the total trace length of the cilium. The tangent angle Chebyshev coefficients ${\hat{\theta }}_{n}(s)$ are defined by Equation (5):

5 $${\hat{\theta }}_{n}(s)=\frac{{k}_{n}}{\pi }\int_{-1}^{1}{{{\rm{d}}}}s\,\frac{\theta (s,t)}{\sqrt{1-{s}^{2}}}{T}_{n}(s)$$

where *k*_0_ = 2 and *k*_*n*_ = 1 otherwise. The 0^*th*^ order coefficient corresponds to a weighted average orientation (Supplementary Fig. 4d) while the 1^*st*^ order coefficient represents a weighted average of the signed curvature.

For each Chebyshev polynomial an approximate wavelength can be assigned to the *n*^*th*^ polynomial as $\frac{1}{2}{\lambda }_{n}=\frac{L}{2(n-1)}\mathop{\sum }_{l=0}^{n-2}\cos \left(\frac{\pi l}{n}+\frac{\pi }{2n}\right)-\cos \left(\frac{\pi (l+1)}{n}+\frac{\pi }{2n}\right)$ which is the mean distance between the extrema scaled by the average cilium length. The factor of 2 in the length scaling is due to the natural interval for Chebyshev polynomials being [ − 1, 1].

### Wavelet analysis and multidimensional scaling analysis

The wavelet transform is performed via the fast Fourier transform using the Morlet wavelet with characteristic frequency 2*π*. The initial and final 20 frames are cropped from the resulting transform to remove boundary artefacts. The transform is performed for each Chebyshev mode trajectory separately.

The dispersion relation is calculated by averaging the wavelet transform for each Chebyshev mode across all time points and chosen experiments. This averaging results in a single vector as a function of frequency. A dispersion heatmap is formed by stacking each time-averaged wavelet vector across Chebyshev modes.

To calculate the multidimensional scaling (MDS) between each instantaneous stacked wavelet vector, the data are randomly distributed into three bins with 1666 time points the same between the bins. Classical MDS is performed separately on each bin using Euclidean distances between the wavelet vectors. Since MDS is only defined up to an affine transformation, the resulting MDS coordinates are then aligned by fitting an affine transform between the overlapping points in each bin to produce a combined MDS embedding^74^.

## Supplementary information

Supplementary Information

Description of Additional Supplementary Files

Supplementary video 1

Supplementary video 2

Supplementary video 3

Supplementary video 4

Transparent Peer Review file

## Data Availability

All datasets necessary to reproduce the work detailed in this manuscript are found at 10.6084/m9.figshare.29941784. Source Data associated with all main and Supplementary Figs. are provided with this paper.

## Code availability

Custom tracking and analysis codes associated with this manuscript are found at 10.6084/m9.figshare.29941784.
