# Supplementary material for: Embodied behavioural complexity in a ciliated microorganism
Source: Nat Commun. 2026 Jul 8;17:8445. doi: 10.1038/s41467-026-75076-8 (PMC13478290; doi:10.1038/s41467-026-75076-8)
Supplement: Supplementary file 2 [file 41467_2026_75076_MOESM2_ESM.pdf]

Supplementary video legends:

**File Name: Supplementary Video 1**

Description: Post-processed video of free-swimming *Pterosperma* cells overlaid with cell trajectories. Imaging was performed under brightfield illumination using a 10x objective.

**File Name: Supplementary Video 2**

Description: Characteristic motile behaviour of *Pterosperma* cells illustrating dynamics in the organism's three behavioural macrostates. Imaging was performed under differential interference contrast microscopy using a 40x (Swim, Reorient) and 63x (Stop) objective.

**File Name: Supplementary Video 3**

Description: Illustrations of the spectral decomposition of ciliary beating across different behavioural states. Microscopy images were obtained using differential interference contrast microscopy and a 40x objective. The corresponding wavelet vector is illustrated for Chebyshev modes 1-9. The position of the ciliary dynamics in the $\mathrm{f} - k$ space is indicated by the red dot.

**File Name: Supplementary Video 4**

Description: Visualisation of the low-dimensional behavioural manifold corresponding to *Pterosperma's* motility behaviour. Microscopy images were obtained using differential interference contrast microscopy and a 40x objective. The red dot indicates the instantaneous position of the organism's dynamics on the low-dimensional behavioural manifold.
