# Supplementary material for: Embodied behavioural complexity in a ciliated microorganism
Source: Nat Commun. 2026 Jul 8;17:8445. doi: 10.1038/s41467-026-75076-8 (PMC13478290; doi:10.1038/s41467-026-75076-8)
Supplement: Supplementary file 3 [file 41467_2026_75076_MOESM7_ESM.pdf]

nature portfolio

Peer Review File

Embodied behavioural complexity in a ciliated microorganism

Corresponding Author: Professor Kirsty Wan

This manuscript has been previously reviewed at another journal. This document only contains information relating to versions considered at Nature Communications.

This file contains all reviewer reports in order by version, followed by all author rebuttals in order by version.

Version 0:

Reviewer comments:

Reviewer #1

(Remarks to the Author)
The revision improves clarity and addresses several of my initial concerns, particularly by (i) clarifying the intended meaning of “embodied” and replacing “aneural”, (ii) removing the “attractor” framing for Fig. 4, and (iii) strengthening the discussion and positioning of the dispersion-relation result. The new SI comparison table is a welcome addition. The rebuttal also includes several additional analyses—and even a new experiment—which I enjoyed reading. The C. elegans dispersion relation was also interesting to see and compare. I thank the authors for the effort in the rebuttal.

While some of this new analysis is deferred to future work, the authors have added useful comparative context to the present manuscript. Although there are still a few aspects I might have approached differently or would have liked to see included in the current manuscript, I respect the authors’ intention to develop these points more fully in a dedicated follow-up paper. Overall, I believe the work is acceptable in its current form.

(Remarks on code availability)

Reviewer #2

(Remarks to the Author)
I thank the authors for their revision and reply to the previous round of comments. The primary substantive critique raised by both referees in the first round concerned the physical and biological origin of the dispersion relation, which both reviewers identified as the paper’s strongest contribution.

In response, the authors have conducted several additional preliminary experiments. In Fig. R1, they review relevant literature on beat-form dynamics in other experimental systems, and they find that a range of systems cluster along a similar diagonal banded structure to their reported dispersion relation. In Fig. R2, they introduce a bead–spring cilia model that exhibits some evidence of a curvature-force relationship. In Fig. R4, they present initial results demonstrating how changes in fluid viscosity (effectively altering mechanical loading) shift the observed behavior space. Taken together, these results suggest a possible shared mechano-chemical mechanism underlying producing a universal dispersion relation across system

However, the authors argue that a fuller mechanistic explanation of this relationship is beyond the scope of the present manuscript and is more appropriately addressed in future work.

I am inclined to agree with the authors argument here. Unpacking notions such as “cognition” in this system likely represents a long-term research program, and the experimental quality and presentation of the current study are very strong enough. Moreover, the discovery of the dispersion relation constitutes a valuable contribution on its own.

Minor:

1. The authors have addressed all minor comments raised by both referees: the the first referee flagged some issues regarding precision of language (eg “attractor,” “excitable”, “aneural”) which the authors addressed. The authors have clarified the questions regarding experiment design.

2. Once the authors have finished exploring this dataset, I hope that the authors will consider making this dataset publicly-available. For example, in neuroscience much data is eventually released on CRCNS.

(Remarks on code availability)

Reviewer #3

(Remarks to the Author)
This manuscript seeks to understand the behaviour of a protist that swims with four cilia. By "behaviour", the authors mean the three motility states - fast swimming (called Swim), slow swimming (called Stop), and a short-lived state between Swim states (called Reorient) - and the transitions between them. And by "understand", they mean how the different behaviours arise from different ciliary beating patterns (wavelength, frequency, and amplitude) and the transitions between these patterns.

It is a credit to the authors that they succeeded in connecting ciliary (organellar) motions occurring on the millisecond timescale with whole-body (organismal) states with lifetimes ranging from 40 ms (Reorient) to ~1 second (Swim) to 1 minute (Stop). The bottom line is that when swimming fast, the cilia move together in a bundle with a symmetric beat. In the Stop state, the cilia splay apart. And during Reorient, the beat becomes transiently asymmetric; when the beat returns to a symmetric one, the swimming direction is different.

The manuscript also explores another level of complexity. This is where it becomes difficult to follow. The transition from Swim to Stop is not all-or-none. Rather, it seems to occur through quasi-discrete changes in frequency and wavelength, with frequency increasing and wavelength (or rather the peak of the wavelength distribution) decreasing. This leads to an interesting "dispersion" plot when the frequency is plotted against the wavenumber (inverse of wavelength) (Figure 3h). This plot is difficult to understand, and it would help if the authors circled regions corresponding to the Swim and Stop states, the transitions Swim->Stop and Stop->Swim, and Reorient (if they are present).

The last part of the manuscript is a "multidimensional (MDS) scaling" of the observed beating patterns. They find that just three dimensions suffice to capture 85% of the variance. When the 200,000 analyzed waveforms are projected onto the three MDS dimensions, they lie predominantly on a surface resembling two wings, with the Stop located at the top of the hinge region (is that right - it would be good to draw a circle/ellipsoid containing 90% of the Stop states). Beautiful examples of swimming cell trajectories are projected onto the surface. However, as usual with these sorts of analyses, it is quite baffling what the three dimensions correspond to. Also, are Swim states (as opposed to the ones transitioning to Stop) tightly clustered on the manifold?

There is a lot of great stuff here, and it should definitely be published. However, this manuscript was difficult to read. The question is not stated, nor is the answer. The writing style is terse. Furthermore, there is a lot of vague language and jargon. For example, from the title and abstract: "embodied" (the opposite of disembodied?), "behavioural complexity", "emerging", "leverage", "dispersion relation" (OED defines it as separation of white light into colours), "elaborate topological structure" (in what way?). "Dynamic transitions in motility behavior are encoded as trajectories in this space": what question does this answer? Also, the molecular basis for these ciliary states is not addressed here. Therefore, the manuscript does not add additional support for (or mechanistic insight into) the last sentence in the Introduction: "the swimming behaviours ... [are] constrained by the nanomechanics of ciliary beat generation": we already knew it.

In summary, the manuscript has reduced the "macroscopic" behavioural states to the "microscopic" dynamic morphological states of the cilia. This is a great achievement!! Just write this story.

(Remarks on code availability)

Version 1:

Reviewer comments:

Reviewer #3

(Remarks to the Author)
The authors have, for the most part, addressed my concerns. Stating the questions makes the manuscript's point clearer. The annotation on the dispersion relation makes it easier for the reader to unpack this figure. Overall, this is an interesting and thought-provoking paper that goes a long way toward connecting molecules to behavior.

(Remarks on code availability)

Open Access This Peer Review File is licensed under a Creative Commons Attribution 4.0 International License, which permits use, sharing, adaptation, distribution and reproduction in any medium or format, as long as you give appropriate credit to the original author(s) and the source, provide a link to the Creative Commons license, and indicate if changes were made.
In cases where reviewers are anonymous, credit should be given to 'Anonymous Referee' and the source.
The images or other third party material in this Peer Review File are included in the article's Creative Commons license, unless indicated otherwise in a credit line to the material. If material is not included in the article's Creative Commons license and your intended use is not permitted by statutory regulation or exceeds the permitted use, you will need to obtain permission directly from the copyright holder.
To view a copy of this license, visit https://creativecommons.org/licenses/by/4.0/

We thank all three reviewers for their careful insights. We are pleased to see that reviewers 1+2 have no further concerns and are now happy to proceed to publication. We provide a point-by-point response to Reviewer 3 below.

Further, considering Reviewers 1+2's comments about the usefulness of the cross-species dispersion data, we have decided to include a **new supplementary figure** (figure 7) based on the figures we provided in the previous rebuttal discussion. This consists of Figure R1a) - which includes the comparison with other sperm data, and the normalised dispersion data from C. elegans (Cohen et al, and Pierce et al), which together help to highlight the cross-species differences between *Pterosperma* and other macroscopic undulatory organisms.

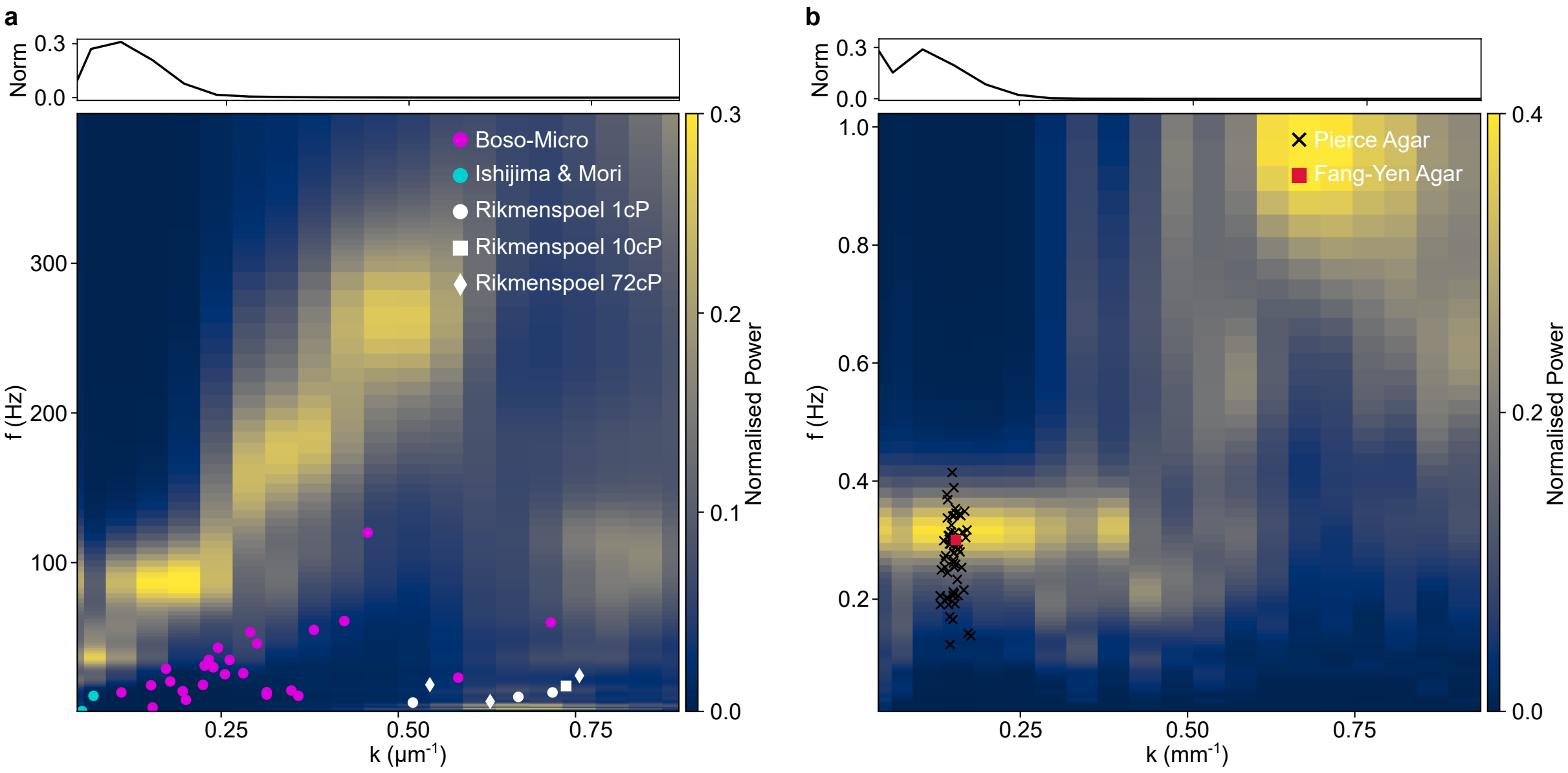

Reviewer #1 (Remarks to the Author):

The revision improves clarity and addresses several of my initial concerns, particularly by (i) clarifying the intended meaning of "embodied" and replacing "aneural", (ii) removing the "attractor" framing for Fig. 4, and (iii) strengthening the discussion and positioning of the dispersion-relation result. The new SI comparison table is a welcome addition. The rebuttal also includes several additional analyses—and even a new experiment—which I enjoyed reading. The C. elegans dispersion relation was also interesting to see and compare. I thank the authors for the effort in the rebuttal.

While some of this new analysis is deferred to future work, the authors have added useful comparative context to the present manuscript. Although there are still a few aspects I might have approached differently or would have liked to see included in the current manuscript, I respect the authors' intention to develop these points more fully in a dedicated follow-up paper. Overall, I believe the work is acceptable in its current form.

We thank the reviewer for their re-review of our work and confirmation that our revision has clearly addressed the previous concerns. As stated above we have added the cross-species dispersion figure to the Supplementary Information to strengthen the comparative context. Yes, we intend to present and discuss the other aspects more thoroughly in the follow-up paper. Stay tuned!

Reviewer #2 (Remarks to the Author):

I thank the authors for their revision and reply to the previous round of comments. The primary substantive critique raised by both referees in the first round concerned the physical and biological origin of the dispersion relation, which both reviewers identified as the paper's strongest contribution.

In response, the authors have conducted several additional preliminary experiments. In Fig. R1, they review relevant literature on beat-form dynamics in other experimental systems, and they find that a range of systems cluster along a similar diagonal banded structure to their reported dispersion relation. In Fig. R2, they introduce a bead–spring cilia model that exhibits some evidence of a curvature-force relationship. In Fig. R4, they present initial results demonstrating how changes in fluid viscosity (effectively altering mechanical loading) shift the observed behavior space. Taken together, these results suggest a possible shared mechano-chemical mechanism underlying producing a universal dispersion relation across system

However, the authors argue that a fuller mechanistic explanation of this relationship is beyond the scope of the present manuscript and is more appropriately addressed in future work.

I am inclined to agree with the authors argument here. Unpacking notions such as "cognition" in this system likely represents a long-term research program, and the experimental quality and presentation of the current study are very strong enough. Moreover, the discovery of the dispersion relation constitutes a valuable contribution on its own.

Minor:

1. The authors have addressed all minor comments raised by both referees: the the first referee flagged some issues regarding precision of language (eg "attractor," "excitable", "aneural") which the authors addressed. The authors have clarified the questions regarding experiment design.

2. Once the authors have finished exploring this dataset, I hope that the authors will consider making this dataset publicly-available. For example, in neuroscience much data is eventually released on CRCNS.

We thank the reviewer for the re-review and summary of the main contributions of our study. We are pleased to see that they are satisfied with our revisions and clarifications, and that they agree with us that a fuller exploration of the other aspects of this work can be addressed in future work.

We can confirm that all waveform data (together with the processed dynamical parameters and the codes) associated with this paper will be made available for other researchers to explore (see Data Availability statement).

Reviewer #3 (Remarks to the Author):

This manuscript seeks to understand the behaviour of a protist that swims with four cilia. By "behaviour", the authors mean the three motility states - fast swimming (called Swim), slow

swimming (called Stop), and a short-lived state between Swim states (called Reorient) - and the transitions between them. And by "understand", they mean how the different behaviours arise from different ciliary beating patterns (wavelength, frequency, and amplitude) and the transitions between these patterns.

It is a credit to the authors that they succeeded in connecting ciliary (organellar) motions occurring on the millisecond timescale with whole-body (organismal) states with lifetimes ranging from 40 ms (Reorient) to ~1 second (Swim) to 1 minute (Stop). The bottom line is that when swimming fast, the cilia move together in a bundle with a symmetric beat. In the Stop state, the cilia splay apart. And during Reorient, the beat becomes transiently asymmetric; when the beat returns to a symmetric one, the swimming direction is different.

The manuscript also explores another level of complexity. This is where it becomes difficult to follow. The transition from Swim to Stop is not all-or-none. Rather, it seems to occur through quasi-discrete changes in frequency and wavelength, with frequency increasing and wavelength (or rather the peak of the wavelength distribution) decreasing. This leads to an interesting "dispersion" plot when the frequency is plotted against the wavenumber (inverse of wavelength) (Figure 3h). This plot is difficult to understand, and it would help if the authors circled regions corresponding to the Swim and Stop states, the transitions Swim->Stop and Stop->Swim, and Reorient (if they are present).

We thank this reviewer for their positive evaluation of our work and suggestions for further improvement. Yes, our intention was indeed to highlight the complexity of the swim to stop transitions, and the unexpectedly nuanced transitions in behaviour in this organism.

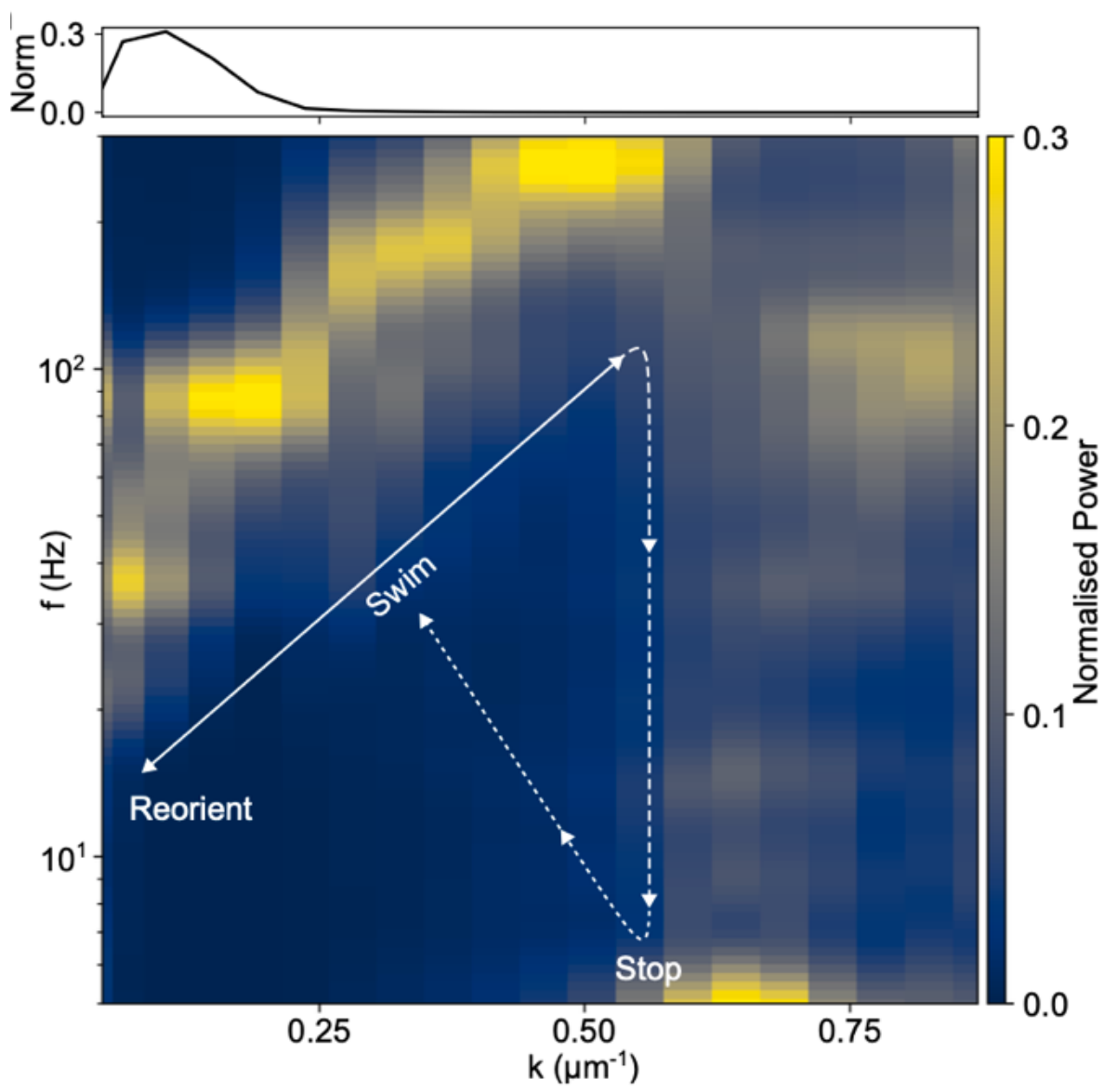

Following the referee's suggestion, we have tried to better explain the interpretation of the dispersion plot in the text. We have added some text labels and arrows to highlight regions in the dispersion plot corresponding to the different states, and allowable transitions between the states. The swim states correspond largely to reversible transitions along the main diagonal. Once the frequency ramps up to a point of no return, the cell commits to a stop behaviour. The cycle 'resets', when the cells rapidly exit the stop state to reappear again on the main 'swim' diagonal.

Note it is customary to plot the frequency against the **wavenumber** in this context, using the wavenumber is preferred over the wavelength, since wavenumber has the convenient interpretation of a 'spatial' frequency, and appears most commonly in mathematical models of ciliary and flagellar beating in the literature.

The last part of the manuscript is a "multidimensional (MDS) scaling" of the observed beating patterns. They find that just three dimensions suffice to capture 85% of the variance. When the 200,000 analyzed waveforms are projected onto the three MDS dimensions, they lie predominantly on a surface resembling two wings, with the Stop located at the top of the hinge region (is that right - it would be good to draw a circle/ellipsoid containing 90% of the Stop states). Beautiful examples of swimming cell trajectories are projected onto the surface. However, as usual with these sorts of analyses, it is quite baffling what the three dimensions correspond to. Also, are Swim states (as opposed to the ones transitioning to Stop) tightly clustered on the manifold?

We thank the reviewer for this observation and suggestions. To help interpret the MDS dimension we have provided some alternative plots in Figure 9 of the SI, where it can be seen that there is a non-trivial relationship between the three MDS dimensions and some more intuitive physical parameters such as swimming speed, beat frequency and wavelength.

In the speed plot we can clearly associate the cluster of points at the top of the hinge to the stop states (very low speed), while from the frequency and wavenumber plots, we can see that increasing wavenumber (decreasing wavelength) corresponds to increasing beat frequency (recovering the main trend from the dispersion plot). Intriguingly, the two different ‘wings’ correspond to different wavenumbers.

We attempted to produce a circle or region containing 90% of the Stop states but found that it was difficult to plot this without obscuring the manifold itself. Instead, see below for a visualisation with all the Stop states coloured in red.

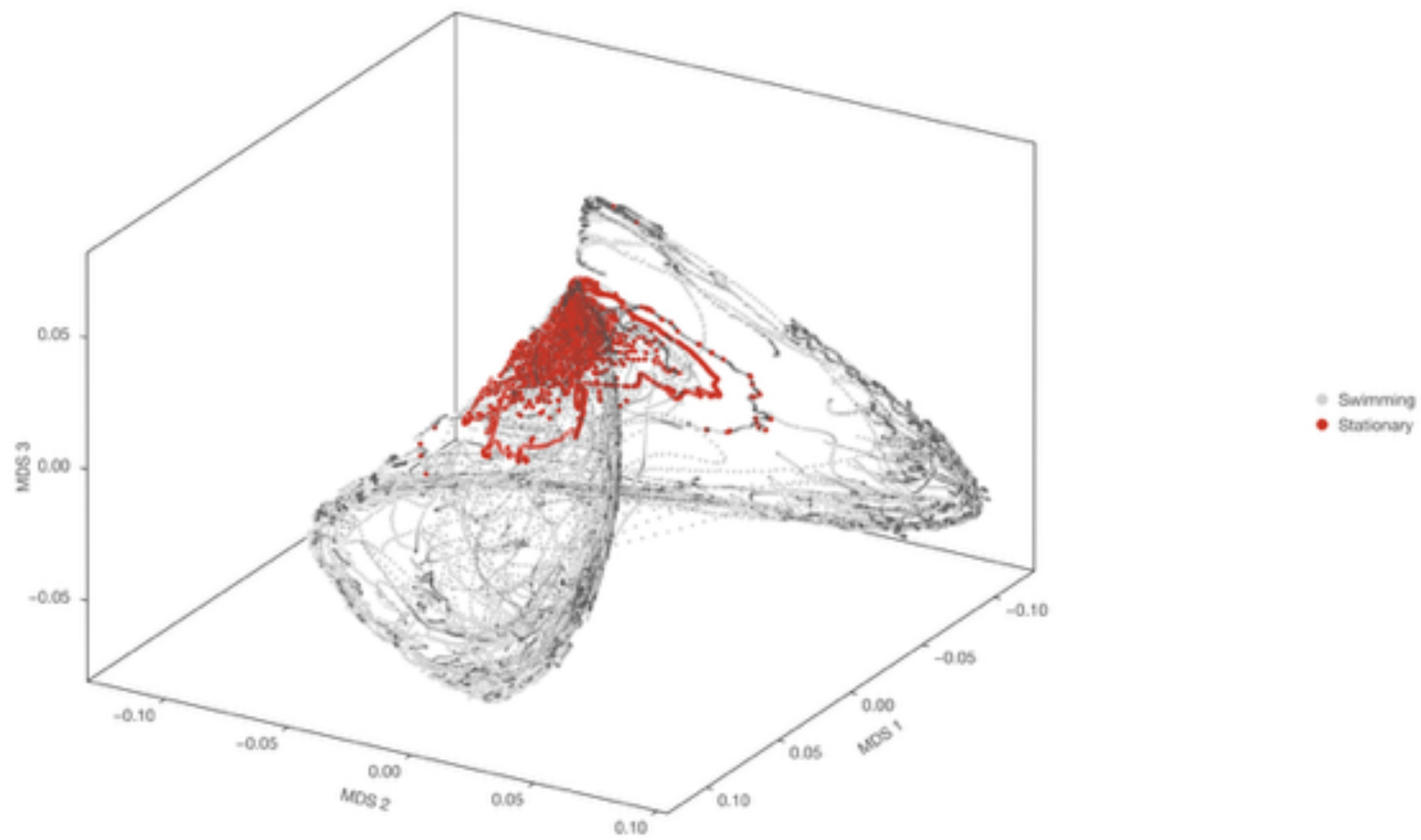

The Swim states are not so much tightly clustered on the manifold but correspond to the bounding envelope where the density of data points is high, in supplementary video 4 you can see some examples of swimming trajectories that traverse smoothly along one edge of the manifold on one side before escaping off it. We remark that the precise shape of the manifold is not so important, more the dimensionality of the space (low given the complexity of the

behaviour displayed), and the fact that this novel analytical approach allows us to visualise how different behavioural sequences traverse the manifold in time.

There is a lot of great stuff here, and it should definitely be published. However, this manuscript was difficult to read. The question is not stated, nor is the answer. The writing style is terse. Furthermore, there is a lot of vague language and jargon. For example, from the title and abstract: "embodied" (the opposite of disembodied?), "behavioural complexity", "emerging", "leverage", "dispersion relation" (OED defines it as separation of white light into colours), "elaborate topological structure" (in what way?). "Dynamic transitions in motility behavior are encoded as trajectories in this space": what question does this answer? Also, the molecular basis for these ciliary states is not addressed here. Therefore, the manuscript does not add additional support for (or mechanistic insight into) the last sentence in the Introduction: "the swimming behaviours ... [are] constrained by the nanomechanics of ciliary beat generation": we already knew it.

In summary, the manuscript has reduced the "macroscopic" behavioural states to the "microscopic" dynamic morphological states of the cilia. This is a great achievement!! Just write this story.

We thank for reviewer for their supportive comments on the novelty and significance of our paper. Considering the criticisms on writing style, we have edited some of the wording in this revision. In fact, this project started with a series of intriguing observations in the absence of a central hypothesis, and the real significance of which was only gradually revealed to us through the subsequent data analysis!

However, we do agree that framing the main questions and answers is extremely helpful for the reader and have clarified the text accordingly. We now state the main questions as, 1) how do we quantify the complex free-swimming behaviours of a single-celled organism, 2) how do these behaviours relate back to the underlying dynamics of the cilia, and 3) what is the space of all possible beat patterns on a cilium?

On reflection, we have kept the title as is. Some terms may be field-specific. Prompted by this reviewer's comments, we also engaged in some further discussions with colleagues from control theory/robotics and concluded that we still do want to highlight the aspect of mechanical or embodied intelligence in a biological system, where the organism's behaviour is governed directly by geometry and physical interactions rather than any 'conscious' neural computation on the part of the organism (see also discussion in the previous rebuttal to reviewers 1 and 2). We have added a few additional relevant citations.

This is what we mean by 'embodied' (a precise and carefully chosen term) and believe this is exactly where our study contributes to an exciting and emerging field. Such true biological examples are rare, precise quantitative studies of such even more so. Therefore, identifying and understanding these phenomena has many valuable implications for bioinspired engineering as well as fundamental biology.

While it is true that we already knew in principle that the nanomechanics of beat generation constrains the beat patterns, we think what this paper adds is **unequivocal experimental proof** for these constraints for the first time, through the discovery of a dispersion relation that constrains the space of possible spatial and temporal beat frequencies on a flagellum, which

both previous reviewers also agreed was the highlight of the paper. This allows direct validation and concrete comparisons to be made between these data and future computational models, which advances the field. The term ‘dispersion relation’ is used commonly in wave theory in physics to indicate the functional relationship between omega (wave frequency) and k (wave number). For consistency with this substantial physics literature, we elect to keep this terminology.

We have removed several instances of the word ‘emerge’/’emergence’, and replace ‘elaborate’ with ‘non-trivial’ topological structure – in reference to the complex shape. We now state in the abstract that: *Dynamic transitions in motility patterns are encoded as trajectories in this space, thus reducing the macroscopic behavioral states to the underlying microscopic dynamics of the cilia*. We also removed several instances of the word ‘leverage’.
